# Prediction of injury risk in Chinese mine rescuers based on single factors and different threshold combinations of FMS and YBT

**DOI:** 10.3389/fpubh.2025.1586179

**Published:** 2025-12-09

**Authors:** Guanghao Yang, Di Gao, Sanjun Yang

**Affiliations:** School of Physical Education, China University of Mining and Technology, Beijing, China

**Keywords:** FMS, YBT, mine rescue personnel, injury risk, injury prediction

## Abstract

**Purpose:**

This study aims to investigate the correlation between FMS and YBT indicators with injuries and to explore a single predictive threshold or combinations of thresholds for practical reference.

**Methods:**

Injury histories of 96 Datong rescue team members were collected via questionnaire, and multiple FMS and YBT indicators were assessed at the same site on different occasions. Data were organized and categorized using Excel and SPSS 29.0. Threshold values derived from relevant literature on comparable occupational groups adopted as reference standards, and statistical analyses (t-test, chi-square) were applied to examine the associations between functional indicators and injury, and to evaluate the validity of both individual and combined threshold criteria.

**Results:**

(1) Injuries primarily concentrated in the lumbar and knee regions, accounting for approximately 50% of total injuries. (2) There were highly significant differences between injured and uninjured groups in FMS scores and YBT bilateral reach distance difference (*p* < 0.01), as well as significant differences in YBT overall score (*p* < 0.05). (3) The injury rate was 76.8% when FMS score < 15, 80% when the YBT lower limb overall score < 95, and 89.39% when the bilateral reach distance difference > 4 cm. (4) When combining the criteria of FMS overall score < 15 and YBT bilateral reach distance difference > 4 cm, the injury rate increased to 90.38%.

**Conclusion:**

(1) The threshold values for the overall scores of FMS and YBT can effectively predict injury risk. In contrast, the YBT bilateral reach distance difference demonstrates superior advantages. (2) The accuracy of combined predictions is higher, with the combination of an FMS 15 and YBT bilateral difference of 4 cm serving as the optimal standard.

## 1 Introduction

The mine rescue team is a crucial component of the national emergency rescue force, specializing in responding to sudden incidents in mines and implementing emergency rescue operations. Their primary responsibilities include controlling the escalation and spread of mining accidents and ensuring the effective evacuation and withdrawal of personnel in hazardous environments, thereby minimizing the loss of life and property (1). Due to the unique nature of their work, mine workers often operate in high-altitude areas while wearing specialized equipment, engaging in prolonged, high-intensity tasks such as rapid crawling, jumping, carrying, and dragging, which places significant physiological and physical demands on rescue personnel (2). The severe conditions associated with their occupation lead to various injury issues. Research indicates that the incidence of sports injuries among national emergency responders is notably high, with common injury sites and characteristics including strains and sprains of the lumbar region and knee joints (3). These injuries severely impact the efficiency of daily rescue operations and can pose life-threatening risks. Therefore, employing scientifically sound predictive methods to understand the probability of injury risk among mine rescue personnel and implementing appropriate intervention measures is critical for enhancing rescue efficiency and ensuring the sustainability of operations.

A considerable body of research has investigated the injury-related mechanisms in occupational rescue populations, with a primary focus on firefighters, and has consistently demonstrated a strong association between impaired physical function and increased occupational injury risk. Mota et al. (4) reported that deficits in neuromuscular capacity are closely linked to reductions in balance and lower-limb stability, thereby elevating the likelihood of falls, trips, and sprains. They further emphasized that functional deficits contribute to injury occurrence predominantly through impaired postural control and diminished lower-limb strength (4). Similar findings were noted by Marciniak et al. (5), who observed that lower scores in dynamic balance assessments among firefighter recruits were significantly correlated with a higher risk of lower-limb injuries. The authors highlighted that such deficits in balance capacity are directly associated with sprains and falls during occupational tasks involving climbing or load carriage (5). Vaulerin et al. additionally found that chronic ankle instability substantially increases the risk of recurrent sprains, underscoring stability deficits as a common injury mechanism (6). Moreover, Buoncristiani et al. (7), Miratsky et al. (8), and Beach (9) argued that asymmetries in lower-limb strength are another critical etiological factor, as such imbalances may lead to uneven joint loading and heightened risk of overload-related injuries. Collectively, these findings indicate that physical functional integrity is intrinsically linked to occupational injury risk, and maintaining a high level of functional competence is essential for injury prevention in rescue populations.

Functional Movement Screening (FMS) and the Y Balance Test (YBT) have been widely employed to assess physical dysfunction and predict sports injuries (10). The emphasis of these two screening tools differs, and their combined assessment can provide more comprehensive data on physical function. The FMS test was originally conceptualized by American corrective exercise specialists Gray Cook and Lee Burton and was formally introduced in the 1990s. It has since been extensively applied in the fields of sports training, rehabilitation, and occupational health. The FMS assesses basic functional deficiencies and impairments related to injury risk through seven prescribed movements, thereby providing a basis for injury prevention (11). In contrast, the YBT is designed to identify the ability to perform specific movements during unilateral support and to assess the degree of asymmetry between sides (12, 13). Originating in the United States, the YBT is a simplified version of the Star Excursion Balance Test (SEBT), reducing the complex lower limb assessment across eight directions to three directions while incorporating upper limb components. This test effectively evaluates balance ability, postural control, neuromuscular control, and the symmetrical stability of both upper and lower limbs (14).

Currently, research on injury risk prediction, both domestically and internationally, has primarily focused on the relationship between single factors and injury prediction in specific sports. In recent years, many scholars have combined both methods for injury prediction, yielding favorable outcomes (15). However, studies concerning injury prediction among mining rescue personnel are scarce and face significant limitations. Few related studies remain focused on single-factor FMS predictive analysis, and there is no consensus on the precise thresholds for predictive indicators. Additionally, no research has addressed the specific thresholds and accuracy comparisons of FMS indicators, YBT indicators, and combined indicators related to injury risk prediction, thus establishing the foundation for the present study. Some researchers have found a correlation between the total FMS score and injury incidence in specialized populations, indicating that when the FMS score is below 15, the probability of injury occurrence significantly increases (16, 17), Comparable findings have also been confirmed in studies involving athletes engaged in high-intensity sports and physically active adult male populations (18–20). Considering the similarities in the nature of work and movement characteristics in emergency rescue groups, this study hypothesizes that an FMS composite score of 15 serves as a threshold for the injury risk of mining rescue teams and aims to validate the correlation between this standard and injury risk.

Currently, there is limited research on the predictive value of YBT for injuries among mining rescue personnel, and no clear high-risk thresholds have been established from the YBT perspective. Several studies, both in China and abroad, have investigated the relationship between YBT-related indicators and injury risk in occupational populations, reaching corresponding conclusions, samples have predominantly focused on firefighters, elite athletes, and police officers. Research indicates that a composite YBT score below 95 or a difference in maximum reach between sides greater than 4 cm can predict a high injury risk (21–29). Therefore, this study attempts to apply these two standards to the analysis of injury risk prediction in mining rescue personnel, exploring and validating the predictive thresholds associated with this population. This research adopts both single testing and combined analysis approaches, aiming to determine suitable injury risk thresholds for mining rescue personnel, establish appropriate standards for high injury risk, and provide theoretical references for implementing adequate preventive measures.

## 2 Methods

### 2.1 Participants

This study selected a total of 96 mine rescuers from the National Mine Emergency Rescue Datong Team (including the Pingwang, Tongfa Dongzhou, Yanya, Sitai, Luyang, Beixinyao, and Madaotou teams) as the subjects for testing. All participants are healthy individuals without acute injuries to the upper limbs, lower limbs, or trunk, ensuring their ability to participate in the tests and minimizing any potential interference from injuries or illnesses in the research outcomes. The subjects are all male, and each participant has previous experience in frontline rescue operations, providing a strong representation for the study. Basic information about the participants is presented in Table 1.

**Table 1:** Basic information of Datong rescue team members.

Number of participants	Age (years)	Height (cm)	Weight (kg)	Years of service
96	34.84 ± 4.31	175.00 ± 5.00	79.19 ± 12.05	11.39 ± 4.87

### 2.2 Experimental testing

(1) FMS test

The Functional Movement Screen (FMS) consists of seven movements, including the overhead deep squat, hurdle step, straight leg lunge, shoulder mobility, active straight leg raise, trunk stability push-up, and rotational stability. Each movement has a maximum score of 3 points, resulting in a total score of 21 points. A score of 3 indicates a standard movement pattern and good physical function; a score of 2 indicates compensatory movements or insufficient range of motion; a score of 1 indicates the inability to complete the movement as required; and a score of 0 indicates pain during the performance of the movement. Additionally, the FMS includes four exclusion tests: the shoulder mobility exclusion test, the reach exclusion test, the ankle mobility exclusion test, and the flexion exclusion test. The FMS composite score is calculated as the sum of the final scores from all individual test items.

The FMS testing was conducted in the spacious indoor venues of the Pingwang and Sitai teams of the Datong Rescue Team from April 1 to April 5, 2023, during the hours of 7:30 a.m. to 12:00 p.m. and 1:30 p.m. to 6:30 p.m., avoiding the team’s regular rest periods. Prior to the testing, all team members received guidance and training on the FMS testing procedures, with the requirement that no participants were to have any acute illnesses or injuries. The FMS test was conducted first, and participants were required to undergo at least 30 min of cool-down prior to the assessment, without engaging in any preparatory activities beforehand. The testing was carried out by personnel with professional testing qualifications.

The testing process involved scoring the quality of movements based on established scoring criteria. The sequence of the testing was as follows: deep squat, hurdle step, straight leg lunge, ankle mobility exclusion test, shoulder mobility, shoulder exclusion test, active straight leg raise, trunk stability push-up, reach exclusion test, rotational stability, and flexion exclusion test. Each movement was tested three times consecutively; if the scores varies, the lowest score is recorded. If the results for both sides differs, the lowest score is taken as the final score for that movement.

(2) YBT test

The complete Y Balance Test (YBT) includes testing components for both the upper and lower limbs, with each component consisting of three testing directions. The upper limb testing directions are: lateral X, medial upper Z, and medial lower Y. The lower limb testing directions are: anterior X, posterior medial Y, and posterior lateral Z. Given the specific injury characteristics and distribution associated with the profession of mine rescue personnel, only the lower limb testing was conducted for this population. The directions were distinguished according to the supporting leg used during the assessments. The composite YBT score is calculated by dividing the sum of the maximum reach distances in the three directions by three times the limb length and then multiplying by 100.

**Figure 1:**
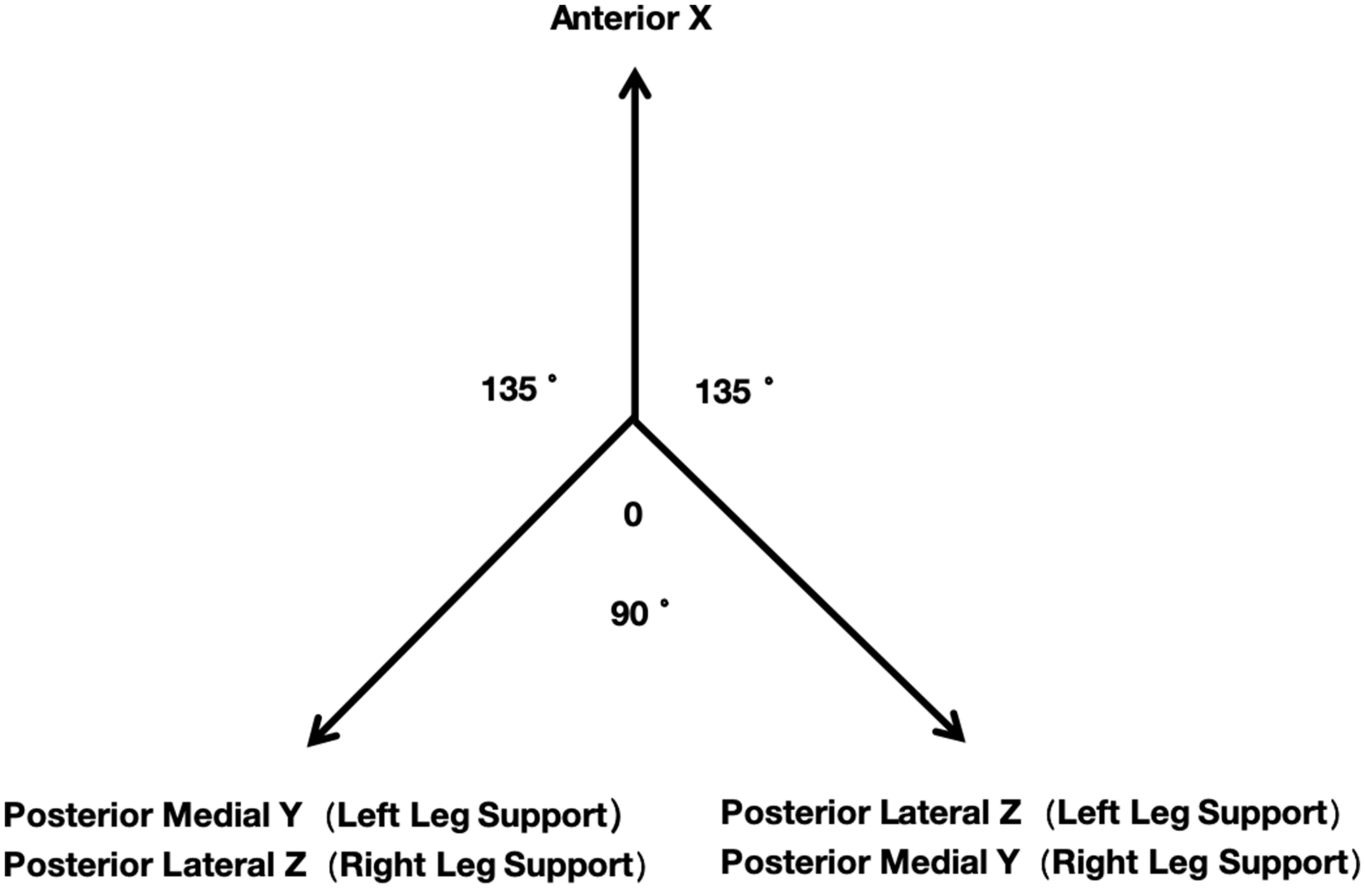
YBT test.

The Y Balance Test (YBT) was conducted at the same location as described in (1). The anterior and posterior tests were scheduled at different time, requiring a minimum gap of 1 h between the two assessments. Prior to testing, qualified personnel provided an introduction and demonstration of the specific procedures and protocols, and no preparatory activities were allowed beforehand. The lengths of both limbs of each participant were measured prior to the test. During the assessment, participants were required to maintain a unilateral support position while using the contralateral limb to push the testing board to the furthest distance. Each direction was measured three times, with the furthest distance touched by the board recorded as the metric for data analysis, accurate to 0.5 cm. Testing was conducted barefoot (see Figure 1).

### 2.3 Questionnaire survey

Considering the practical requirements for investigating the overall injury status of mine rescue personnel, a questionnaire was designed based on relevant research regarding injury assessment in mine rescue teams (30). The questionnaire encompassed various aspects, including personal demographic information, injury locations, types of injuries, sports activities that caused the injuries, timing of injury occurrences, management following injuries, and recovery status. The questionnaire aimed to collect data on up to three injury incidents experienced by rescue team members within the past 5 years. If a participant had more than three injuries, only the three most severe incidents were documented. Upon completion of the questionnaire design, experts in physical training and emergency rescue validated the questionnaire for its effectiveness (Appendix A).

All questionnaires were completed and collected on-site, with professional personnel available to address any questions from the rescue personnel during the filling process. Questionnaires that took less than 60 s to complete or had a missing data rate exceeding 10% were not considered valid. A total of 96 questionnaires were distributed, with no questionnaires excluded, resulting in the collection of 96 valid responses. The response rate for valid questionnaires is 100% (Appendix B).

To ensure the reliability of the questionnaire results, a retest of the injury history questionnaire was conducted 2 weeks later with a total of 51 team members who had reported an injury. The questionnaire consisted of 6 questions, and participants answered according to the same procedures and protocols. Subsequently, the responses to the 6 questions were analyzed for test–retest reliability, and the average test–retest reliability coefficient (R) was calculated. A total of 51 questionnaires were distributed for the retest, with 50 returned. After excluding 3 invalid questionnaires, 47 valid retest questionnaires remained, resulting in a response rate of 94.00%. As shown in Table 2, the overall test–retest reliability of the questionnaire was 0.876, with each individual question demonstrating a test–retest reliability higher than 0.85. This indicates a high level of reliability, suggesting that the data is both authentic and reliable, making it suitable for subsequent research and analysis.

**Table 2:** Test–retest reliability consistency check.

Question number	Number of questionnaires	Number of consistent answers	Consistency rate
1	47	43	0.915
2	47	41	0.872
3	47	42	0.894
4	47	40	0.851
5	47	40	0.851
6	47	41	0.872
	Average	41.17	0.876
		R = 0.876	

### 2.4 Statistical analysis

Statistical analyses of the experimental data were conducted using Microsoft Excel and SPSS 29.0. Descriptive statistics were performed for the basic characteristics of the rescue team members, including height, weight, age, and injury data. Independent sample t-tests were conducted to compare the FMS composite scores, YBT composite scores, and bilateral differences in YBT performance between participants with and without a history of injury. Chi-square tests were performed for the following categories: FMS scores ≤15 and >15, YBT scores ≤95 and >95, bilateral stretch distance differences ≤4 cm and >4 cm, as well as combinations of these criteria. A significance level of *p* < 0.05 was considered statistically significant, while *p* < 0.01 was regarded as highly significant.

### 2.5 Study design

**Figure 2:**
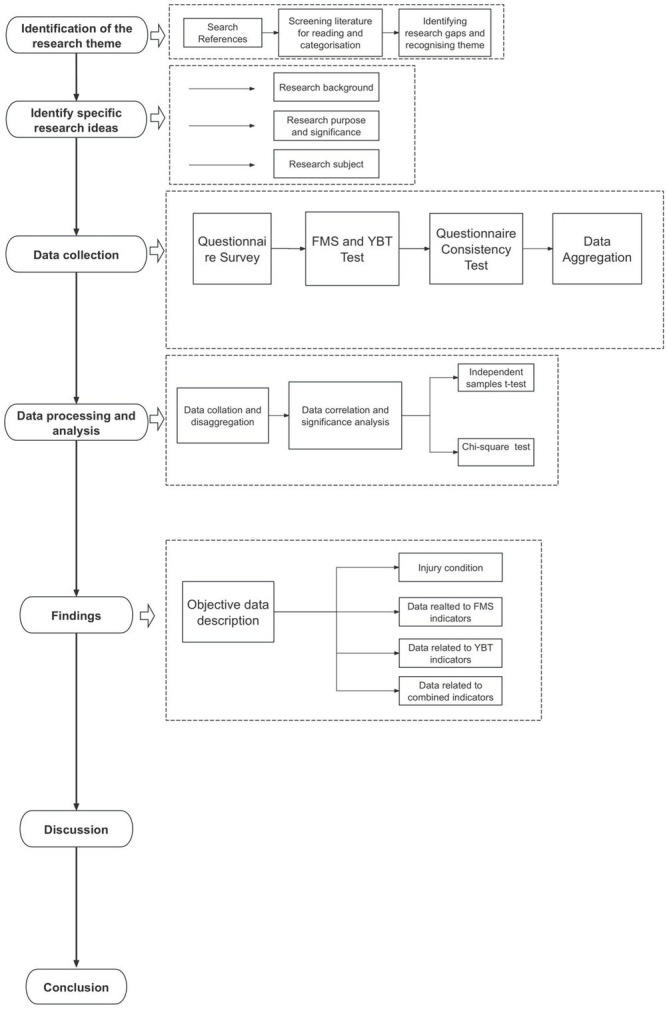
Writing flowchart.

For an overview of the general process, see Figure 2.

## 3 Results

### 3.1 Investigation results of injury situations of the Datong mine emergency rescue team

According to statistics, among the 96 members of the Datong Mine Rescue Team, 68 individuals have sustained injuries, resulting in an injury rate of 71.00%. This indicates a high risk of injury within this population, warranting focused attention. Within the injured group, a total of 89 injuries were reported, averaging 1.3 injuries per person. Analysis of injury sites reveals that the lumbar region is the most frequently injured area, with 39 cases, accounting for 43.82% of all injuries (Table 3). The knee joint is the second most common site of injury, comprising 10.11% of the total. Additionally, injuries to the elbow, shoulder, upper arm, forearm, and hand are also significant; however, the overall proportion of injuries in these areas is notably lower compared to lumbar injuries.

**Table 3:** Statistical results of injury incidents in the Datong mine emergency rescue team.

Injury site	Number of injuries	Percentage (%)
Lumbar	39	43.82
Shoulder Joint	5	5.61
Knee Joint	9	10.11
Upper Arm and Forearm	5	5.62
Elbow Joint	5	5.62
Wrist Joint	3	3.37
Hand	7	7.87
Chest and Back	2	2.25
Calf	1	1.12
Ankle Joint	3	3.37
Thigh(anterior)	2	2.25
Foot	4	4.49
Neck	2	2.25
Head	2	2.25

### 3.2 Distribution of FMS total scores in the Datong mine rescue team

The statistical analysis of the distribution of the FMS composite scores for the Datong team (Table 4) reveals a wide range of scores. The lowest score recorded was 1, achieved by 2 individuals (2.08% of the participants), while the highest score reached 18, attained by 1 individual (1.04%). The most frequent score was 10, with a total of 17 participants (17.71%). Overall, the distribution of scores exhibits an inverted U-shaped trend, with the majority of FMS scores concentrated in the range of 8 to 15, accounting for 72 individuals (75.00%).

**Table 4:** The distribution of FMS composite scores for the Datong rescue team.

	Score	Count (people)	Percentage (%)
FMS composite score	1	2	2.08
	3	1	1.04
	4	1	1.04
	5	1	1.04
	6	9	9.38
	7	2	2.08
	8	8	8.33
	9	9	9.38
	10	17	17.71
	11	5	5.21
	12	7	7.29
	13	6	6.25
	14	11	11.46
	15	9	9.38
	16	4	4.17
	17	3	3.13
	18	1	1.04
Total		96	100

### 3.3 Statistical results of FMS composite scores and injury conditions

Table 5 presents the statistical analysis of the FMS composite scores for rescue team members with and without injuries. From the preliminary data, it can be observed that the FMS score for the non-injured group (14.64 ± 2.53) is roughly higher than that of the injured group (10.40 ± 3.88). An independent samples t-test was conducted on the two groups, revealing a result of *p* < 0.01, indicating a significant difference between the two datasets. Therefore, it can be concluded that the FMS scores for the non-injured group of the Datong rescue team are higher than those of the injured group.

**Table 5:** Statistical results of FMS composite scores for injured and non-injured groups.

Injury status	Count (people)	FMS composite score	*P*
Injured	68	10.40 ± 3.88	*P* < 0.01**
Non-injured	28	14.64 ± 2.53	

**Very significant difference (*p* < 0.01).

As shown in the statistical results in Table 6, there were 82 individuals in the FMS < 15 group and 14 individuals in the FMS ≥ 15 group. In the FMS < 15 group, 63 individuals sustained injuries, while 19 did not, yielding an injury rate of 76.8%. In the FMS ≥ 15 group, 5 individuals sustained injuries and 9 did not, resulting in an injury rate of 35.7%.

**Table 6:** Statistical results of injury status and predefined FMS score threshold intervals.

Injury status	FMS < 15	FMS ≥ 15	X^2^	*P*
Injured	63	5	9.785	*P* < 0.01**
Non-injured	19	9		
Total	82	14		
Injury rate (%)	76.8	35.7		

**Very significant difference (*p* < 0.01).

To verify the relationship between the predefined score threshold and injury incidence rate, a chi-square test was performed on the statistical data. The results yielded a chi-square value of 9.785 with *p* < 0.01, indicating a significant difference in injury rates between the two scoring intervals.

### 3.4 Statistical results of YBT performance and injury status

Independent sample t-tests were conducted for YBT-related data. Table 7 presents the statistical results of YBT data in relation to injury status. The results indicate a significant difference in composite YBT scores between the injured and uninjured groups (P1 < 0.05), with the injured group showing a lower composite YBT score (95.07 ± 9.60) compared to the uninjured group (99.80 ± 7.11). Furthermore, a highly significant difference was found in bilateral lower limb reach difference between the groups (P2 < 0.01), with the injured group showing a greater reach difference (10.71 ± 7.04) compared to the uninjured group (5.25 ± 4.27).

**Table 7:** Statistical Results of YBT Test Data and Injury Status.

Injury status	Number of individuals	YBT lower limb composite score	P1	YBT bilateral lower limb reach difference	P2
Injured	68	95.07 ± 9.60	*P* < 0.05*	10.71 ± 7.04	*P* < 0.01**
Non-injured	28	99.80 ± 7.11		5.25 ± 4.27	

*Significant difference (*p* < 0.05); **very significant difference (*p* < 0.01).

As shown in Tables 8, a total of 70 individuals were in the YBT composite score < 95 group, significantly higher than the 26 individuals in the YBT composite score ≥ 95 group. Within the YBT composite score < 95 group, there were 56 individuals with injuries, resulting in an injury rate of 80%. In contrast, the YBT composite score ≥ 95 group had 12 individuals with injuries, yielding an injury rate of 46.2%. These results indicate a obvious difference in injury rates between the two groups.

**Table 8:** Statistical results of injury status on predefined YBT lower composite score threshold intervals.

Injury status	YBT lower composite score < 95	YBT lower composite score ≥ 95	X^2^	*P*
Injured	56	12	10.512	*P* < 0.01**
Non-injured	14	14		
Total	70	26		
Injury rate (%)	80.0	46.2		

**Very significant difference (*p* < 0.01).

A Chi-square testing of the statistical data yielded an X^2^ value of 10.512 with *p* = 0.01 < 0.05. Therefore, it can be concluded that there is a significant difference in injury status between the two groups.

As shown in Table 9, in the YBT lower-limb reach difference interval of >4, there were 66 individuals, of whom 59 experienced injuries, resulting in an injury rate of 89.39%. In the ≤4 interval, there were 30 individuals, with 9 experienced injuries, yielding an injury rate of 30.00%. The injury rates between the two intervals differ significantly. Further analysis using Chi-square testing yielded X^2^ = 32.217 with *p* < 0.01, indicating a highly significant difference in injury incidence between the two intervals.

**Table 9:** Statistical results of injury status based on YBT lower-limb reach distance difference intervals.

Injury status	YBT lower-limb reach distance difference > 4	YBT lower-limb reach distance difference ≤ 4	*X* ^2^	*P*
Injured	59	9	32.217	*P* < 0.01**
Non-Injured	7	21		
Total	66	30		
Injured Rate(%)	89.39	30.00		

**Very significant difference (*p* < 0.01).

### 3.5 Statistical results of injury status and combined threshold indicators of FMS and YBT

Although FMS and YBT threshold indicators can predict injury risk with high probability, there are still instances where individuals with good lower-limb symmetry and functionality sustain injuries, while others with poorer functionality remain uninjured. Therefore, to enhance the accuracy of injury risk prediction and identify optimal standards for predicting injuries, combining different threshold indicators for analysis is an effective approach.

Table 10 presents a combined statistical analysis of injury status based on the threshold intervals of FMS and YBT composite scores. The results indicate that the group not meeting both thresholds had the highest number of injuries, with 26 individuals and an injury rate of 92.86%, the highest among the four groups. In contrast, the group meeting both thresholds had the lowest injury rate, at 7.69%. Additionally, the table shows a slight decrease in injury rate when one of the two threshold standards is met, with a relatively higher risk of injury associated with unmet FMS composite scores in both combinations.

**Table 10:** Statistical results of injury status based on combined threshold intervals of FMS and YBT composite scores.

Injury status	FMS < 15		FMS ≥ 15		X^2^	*P*
	YBT lower composite score < 95	YBT lower composite score ≥ 95	YBT lower composite score < 95	YBT lower composite score ≥ 95		
Injured	26	38	3	1	35.591	*P* < 0.01**
Non-injured	2	12	2	12		
Total	28	50	5	13		
Injury rate (%)	92.86	76.00	60.00	7.69		

**Very significant difference (*p* < 0.01).

Chi-square testing on the data in Table 10 resulted in X^2^ = 35.591, which exceeds the X^2^ values observed in single-threshold tests, indicating a highly significant difference in injury rates among the four conditions. This combined analysis is statistically more meaningful than single-criteria analysis. After conducting pairwise combination tests of the four conditions, significant differences were still observed (*p* < 0.05).

Table 11 shows the results of the combined analysis of FMS composite score threshold intervals and YBT lower-limb reach distance difference threshold intervals. The statistical results indicate that in the case where both threshold criteria are not met, there were 52 individuals, of whom 47 experienced injuries, resulting in an injury rate of 90.38%. In contrast, the group meeting both criteria had the lowest injury rate at 10%. When one of the indicators met the required threshold, the injury rate decreased accordingly.

**Table 11:** Statistical results of combined analysis of FMS composite score and YBT lower-limb reach distance difference threshold intervals.

Injury status	FMS < 15		FMS ≥ 15		X^2^	*P*
	YBT lower-limb reach difference > 4	YBT lower-limb reach difference ≤ 4	YBT lower-limb reach difference > 4	YBT lower-limb reach difference ≤ 4		
Injured	47	8	9	2	43.542	*P* < 0.01**
Non-Injured	5	3	4	18		
Total	52	11	13	20		
Injury rate (%)	90.38	72.73	69.23	10.00		

**Very significant difference (*p* < 0.01).

Chi-square testing on the injury status across the four conditions revealed X^2^ = 43.542 (which is higher than the X^2^ values observed in single-factor tests), with *p* < 0.01, indicating a significant difference in injury incidence across the four conditions. This combined analysis provides more statistical significance compared to single-threshold analysis.

Based on the combined statistical results from Tables 10, 11, it can be concluded that the two threshold combinations provide more statistically significant predictions compared to single-threshold predictions. Among the two threshold combinations, the combination of FMS composite score and lower-limb reach distance difference resulted in an X^2^ value of 43.542, which is higher than the X^2^ value of 35.591 obtained from the combination of FMS and YBT composite scores.

## 4 Discussion

### 4.1 Injury statistics

The survey data of this study show that the Datong Mine Rescue Team has a high injury rate, with 68 out of 96 team members having a history of injury, resulting in an injury rate of 70.83%. The lumbar region is the most commonly injured area, with 39 reported injuries, accounting for 43.82% of the total injury incidents. These injuries are primarily chronic, caused by long-term, high-intensity work. The high injury rate is closely related to the team’s daily training programs and professional characteristics. It should be noted that injury data collected through self-recall and self-report may lack sufficient rigor, whereas the inclusion of official medical documentation would facilitate more accurate and reliable statistical reporting. In addition, the exclusive inclusion of male participants and the narrow sampling frame may render the results biased and incidental, thereby limiting the generalizability of the conclusions, Future studies should expand the sample coverage in terms of sex, age, and occupational categories.

Firstly, statistical data on the training activities leading to injuries (Appendix C) show that most of the team’s training methods exert significant pressure on the lumbar region. In particular, the drag-and-pull training is identified as the primary cause of lumbar injuries, with 31 reported cases, accounting for 34.8% of all injuries. The repetitive downward pulling motion generates eccentric loads, which, when applied over a long period, easily lead to chronic injuries. Marras et al. (31) further supported this view, indicating that prolonged, high-intensity repetitive movements place significant eccentric load pressure on the lumbar region, leading to the accumulation of injuries. Additionally, a study by Gordon et al. (32) on firefighters and rescue personnel found that repetitive drag-and-pull training overloads the lumbar region, significantly increasing the risk of chronic injuries.

Weighted running is another significant cause of lumbar injuries, likely related to the lumbar region serving as the main support point under load. Long-duration weighted running exercises can easily lead to chronic strain on the lower back. Hoffman and Kang (33) supported this view, stating that the sustained pressure from weighted running and other weight-bearing activities causes fatigue accumulation over time, ultimately leading to chronic injuries. Similarly, carrying and weighted uphill training exert direct pressure on the lumbar region. Kim et al. (34) found in their study of rescue and emergency response occupations that carrying and climbing actions, when repeatedly performed in poor postures, significantly increase the load on the lumbar region, leading to chronic injuries. These high-risk training activities are prevalent in the team’s daily regimen and are likely a major contributing factor to the consistently high injury rate.

Furthermore, team members often engage in prolonged, high-intensity weight-bearing tasks such as carrying, hauling, and dragging in harsh environments. The lack of proper body mechanics and force application habits, combined with physical and mental fatigue, increases the risk of acute injuries, which can gradually develop into chronic ones (35). Choi et al. (36) pointed out that high-intensity weight-bearing work in extreme environments not only increases physiological stress but also significantly raises the probability of acute injuries. The cumulative effect of both physical and mental fatigue may further contribute to the high incidence of chronic injuries.

Based on the above situation, improving the injury status of rescue team members should focus on the following aspects: (1) Strengthen the scientific training model for rescue team members, especially by reasonably selecting training equipment and technical movements to avoid excessive pressure on the lumbar region caused by high-frequency eccentric loading movements. At the same time, wearing protective gear properly and improving lumbar strength and endurance will also help enhance their ability to resist injuries. (2) Optimize the training program and reasonably control the frequency and intensity of high-load training programs to reduce the occurrence of chronic lumbar injuries.

### 4.2 Single threshold indicators of FMS and YBT

In this study, the FMS-related statistical results indicate that the overall FMS composite score of the group with a history of injury (10.40 ± 3.88) is lower than that of the group without a history of injury (14.64 ± 2.53). Using an FMS composite score threshold of 15 revealed a significant difference in injury incidence between groups (*p* < 0.01), suggesting that an FMS score of 15 can serve as an effective standard for injury prediction in this cohort. Similar conclusions were reached by Qiao and Zhang (16) and Chu (17) in injury prediction studies on groups with similar occupational characteristics. This threshold is consistent with the findings of Kiesel et al. (37) and Chorba et al. (38), who also identified a score of 15 as a valid injury risk assessment threshold for high-physical-demand groups, such as firefighters and security personnel. Furthermore, studies by Shore et al. (39) and Kollock et al. (40) on injury risk prediction in other populations have also indicated that a predictive baseline of 15 points demonstrates strong applicability for injury risk assessment.

The predictive validity of the same FMS threshold has also been confirmed in athlete populations. Schneiders et al. (41) found that athletes with an FMS score below 15 had a significantly increased probability of injury (OR = 2.74, *p* < 0.01). Similarly, Filipa et al. (42) reported that individuals scoring below 15 had a higher risk of injury in a study on adolescent athletes. These studies consistently support the predictive efficacy of an FMS threshold of 15 across diverse populations, further validating the generalizability of the findings in this study.

In this study, YBT-related statistical results indicated that the YBT composite score for the injured group (95.07 ± 9.60) was lower than that of the non-injured group (99.80 ± 7.11). Additionally, the bilateral reach distance difference in the injured group (10.71 ± 7.04) was significantly higher than in the non-injured group (5.25 ± 4.27). Injury risk analysis using a threshold of a YBT score of 95 and a bilateral reach distance difference of 4 cm showed both metrics were significantly associated with injury risk (*p* < 0.01), suggesting that both YBT thresholds can serve as effective injury risk predictors in this population. Similar findings were observed in injury risk studies involving similar populations by Su et al. (21), Lehr et al. (22), Fusco et al. (23), and Nakagawa et al. (24). Furthermore, research by Xie et al. (43) and Borghuis and Stein (44) on YBT injury risk predictors aligns with these standards, identifying both bilateral reach difference and composite scores as sensitive indicators of injury risk in physically demanding occupations. Additionally, studies by Taylor et al. (45) and Plisky et al. (46) found a significantly higher injury risk among athletes and military personnel with larger bilateral reach discrepancies, with a discrepancy greater than 4 cm notably increasing the probability of injury among military and manual labor groups. Shin et al. (47) and Kovčan et al. (48) further demonstrated that individuals with lower YBT composite scores have a higher injury risk in high-physical-demand occupations, but specific threshold scores for this metric remain underexplored.

The predictive value of the three threshold standards of FMS and YBT for injury risk has been effectively validated within the mine rescue team, and these standards demonstrate good generalizability. While injury prediction research for this specialized group remains limited, this study attempts to bridge part of this gap by establishing multi-dimensional injury prediction standards, which serves as its innovative aspect. A deeper analysis of the correlation between these threshold standards and injury incidence revealed that, among the three indicators, the YBT bilateral reach difference threshold of 4 cm, when analyzed with injury data, demonstrated the highest statistical significance, with an X^2^ value of 32.217, surpassing the other two standards. This finding implies that, within the mine rescue population, the YBT bilateral reach difference threshold may offer superior predictive power for injury risk relative to both FMS and YBT composite scores. Consequently, it is recommended that in future risk assessments focusing on single-indicator prediction, the YBT bilateral reach difference should be prioritized as the primary injury risk predictor for mine rescue personnel.

### 4.3 FMS and YBT combined threshold indicators

The data results from the combined analysis in this study indicate that the statistical results of the two combined scenarios (X^2^ = 43.542 and X^2^ = 35.591) are significantly higher than any of the single-factor analysis results (X^2^ = 32.217, X^2^ = 10.512, and X^2^ = 9.785), suggesting that combined analysis can more accurately identify potential injury risks. This conclusion is also supported by other studies. Myer et al. (49) and Eckart et al. (28) found that using a combination of FMS and YBT in athlete populations effectively identifies high-risk individuals and significantly improves prediction accuracy. In occupational injury risk prediction, Marras et al. (31) also noted that combining multiple fitness screening tools, such as FMS and YBT, helps optimize predictive models and enhances specificity. Gordon et al. (32) further supported this approach, stating that the combination of FMS and YBT not only screens for high-risk individuals but also helps reduce injury rates through corresponding functional improvement training.

In the specific combined analysis, the combination of FMS composite score and YBT lower limb bilateral reach distance difference shows greater statistical significance (X^2^ = 43.542 compared to X^2^ = 35.591) than the other combination, indicating that this combination is more advantageous in predicting injury risk in the mining rescue population. Particularly, the situation where both threshold values are unmet yields the highest injury prediction accuracy, making it an optimal standard for predicting injury risk in mining rescue personnel. In practical application, this tool can be used to provide early warning alerts for populations with potential injury risks and offer corresponding functional improvement interventions, thereby reducing the injury incidence in this group and ensuring the sustainability of occupational activities. It is noteworthy that, although the combined application of the two threshold standards demonstrates greater accuracy in injury risk association, challenges remain in terms of standardized and specialized testing procedures, data reliability, and potential complexities in statistical analysis and data aggregation. Moreover, equipping each unit with two sets of advanced functional testing devices may impose considerable costs. From this perspective, the adoption of a single functional screening tool may offer greater practicality and cost-effectiveness, whereas the combined use of thresholds could yield superior predictive outcomes if resources and operational conditions permit.

This study identified several threshold indicators associated with occupational injury risk through mathematical analysis. However, a notable limitation is that the cross-sectional design does not allow for causal inference. Thus, the observed associations between threshold indicators and injury risk should not be interpreted as universally generalizable results but may serve as auxiliary references in practical applications. Future research with larger, more diverse cohorts and longitudinal or interventional designs is warranted to validate these threshold indicators and establish causal relationships.

## 5 Conclusion

1. Injuries are common in the mining rescue population, with injuries primarily concentrated in the lumbar and knee joints. Among these, the lumbar region is the most vulnerable to injury, followed by the knee joint.
2. In single-factor prediction, the FMS composite score of 15, the YBT lower limb composite score of 95, and the bilateral reach distance difference of 4 cm in the lower limbs can all serve as effective standards for assessing the injury risk rate in this group. Among these, the 4 cm threshold for the YBT bilateral reach distance difference is more advantageous than the other two in predicting injuries.
3. The accuracy of the combined prediction is significantly higher than that of any single-threshold prediction. The combination of the FMS composite score and the YBT lower limb bilateral reach distance difference threshold standard is the optimal standard for predicting injury risk in the mining rescue team.

## Data Availability

The raw data supporting the conclusions of this article will be made available by the authors, without undue reservation.
