# Supplementary material for: Prediction of injury risk in Chinese mine rescuers based on single factors and different threshold combinations of FMS and YBT
Source: Front Public Health. 2025 Dec 9;13:1586179. doi: 10.3389/fpubh.2025.1586179 (PMC12746483; doi:10.3389/fpubh.2025.1586179)
Supplement: Supplementary file 1 [file Data_Sheet_1.docx]

**Appendix A**

**Validity Assessment of the Questionnaire**

**Dear Experts/Mine Rescuers,**
Greetings!

I am a master's student at China University of Mining and Technology (Beijing), Class of 2023. Currently, I am conducting research titled "Prediction of Injury Risk in Chinese Mine Rescuers Based on Single Factors and Different Threshold Combinations of FMS and YBT."

To support this research, I have designed a questionnaire investigating the injury history of mine rescuers. Kindly evaluate whether the validity of this questionnaire meets the required standards. The evaluation consists of five levels: “Reasonable,” “Relatively Reasonable,” “Average,” “Less Reasonable,” and “Unreasonable.” Please mark “√” in the corresponding category that best reflects your assessment of the questionnaire. Additionally, we welcome your valuable suggestions and comments.

Thank you for your support of this study. I sincerely express my gratitude for your guidance and assistance!

**Graduate Student: Guanghao Yang**

**Questionnaire Evaluation Table**

| Evaluation of the Questionnaire | Reasonable | Relatively  Reasonable | Average | Less  Reasonable | Unreasonable |
| --- | --- | --- | --- | --- | --- |
| Expert Evaluation |  |  |  |  |  |
| Comments or Suggestions |  |  |  |  |  |

**Appendix B**

**Questionnaire**

**Personal Information**

1. Your Name:
2. Your Employee ID:
3. Your Age:
4. Your Weight:
5. Years of Experience in Mine Rescue Work:
6. Have you ever experienced an injury within the scope of this questionnaire? (Single choice. If you select A, please continue; if B, the questionnaire ends.)
   A. Yes
   B. No
7. How many injuries have you experienced? (Single choice)
   A. 0 times
   B. 1 time
   C. 2 times
   D. 3 times

### **Injury History Survey Form**

1. **Which part of your body was injured?** (Single choice)
   A. Head
   B. Neck
   C. Chest and Back
   D. Shoulder
   E. Upper Arm and Forearm
   F. Elbow
   G. Wrist
   H. Hand
   I. Lower Back
   J. Groin
   K. Front Thigh
   L. Back Thigh
   M. Calf
   N. Knee
   O. Ankle
   P. Foot
   Q. Other

**2. During which of the following activities did the injury occur?** (Select one option from ①, ②, or ③)

① **Physical Training**
A. Sprinting
B. Middle-distance running
C. Long-distance running
D. Push-ups
E. Sit-ups
F. Pull-ups
G. Parallel Bars
H. Obstacle Running
I. Frog Jumping
J. Zigzag Running
K. Weighted Running
L. Other

② **Basic Mine Rescue Operations**
A. Scaling a 100-meter Barrier
B. Putting on and Using Rescue Gear
C. Using Air Breathing Apparatus
D. Transporting Supplies over 400 Meters
E. Hooking and Climbing a Building
F. Connecting Fire Hoses
G. Using a Traction Device
H. Other

③ **Mine Rescue Specialized Training**
A. Operating Specialized Vehicles
B. Rope Climbing
C. Weighted Uphill Marching
D. Firefighting Training
E. Carrying Training
F. Dragging Training
G. Search Training
H. Other

**3. What type of injury did you experience?** (Single choice)
A. Skin Abrasion
B. Contusion
C. Sprain
D. Strain
E. Dislocation
F. Fracture
G. Overuse Injury
H. Other

**4. When did this injury occur?** (Single choice)
A. During Training
B. During Emergency Rescue Missions
C. During Competitions
D. Other

**5. How was the injury treated?** (Single choice)
A. Hospitalized
B. Treated at a Hospital Outpatient Clinic
C. Treated by a Teammate
D. Self-treated

**6. What is your current recovery status?** (Single choice)
A. Fully Recovered
B. Still Experiencing Pain
C. Limited Mobility
D. Loss of Function

**Appendix C**

**Statistics on Training Activities Causing Lower Back Injuries in Datong Team Members**

**
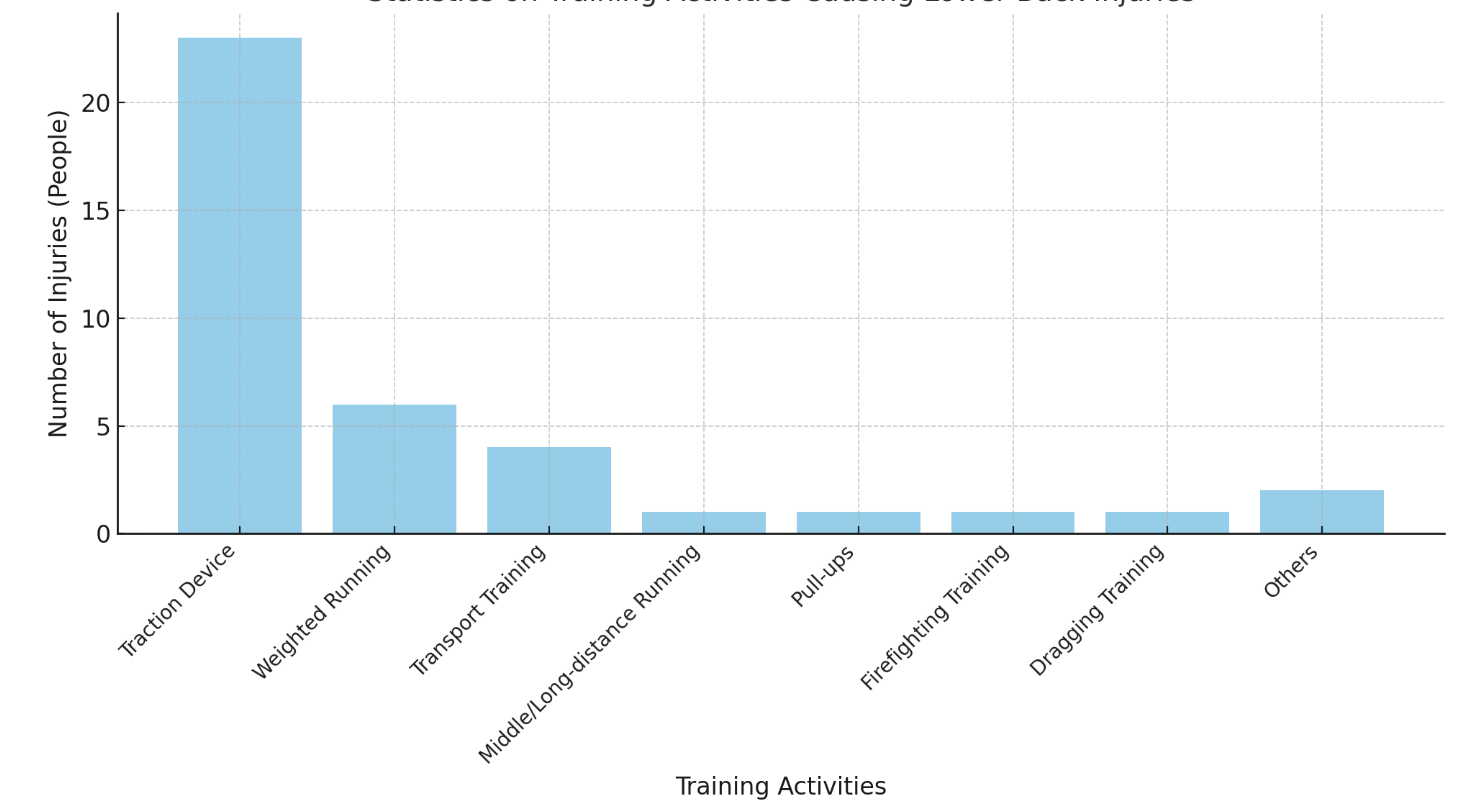
**
